# DDI-HierPred: An Artificial Intelligence-Based Hierarchical PK/PD Platform for Drug–Drug Interaction Prediction

**DOI:** 10.3390/pharmaceutics18081042

**Published:** 2026-08-21

**Authors:** Mebarka Ouassaf, Bader Y. Alhatlani

**Affiliations:** 1 Group of Computational and Medicinal Chemistry, LMCE Laboratory, University of Biskra, Biskra 07000, Algeria; 2 Unit of Scientific Research, Applied College, Qassim University, Buraydah 52571, Saudi Arabia

**Keywords:** drug–drug interaction, machine learning, linear SVM, XGBoost, morgan fingerprints, pharmacokinetic interaction, pharmacodynamic interaction, hierarchical classification, web platform

## Abstract

**Background/Objectives:** Pharmacokinetic and pharmacodynamic drug–drug interactions are major determinants of drug safety in polypharmacy, with potential consequences including reduced therapeutic efficacy, altered drug exposure, and increased adverse effects. This study presents DDI-HierPred, an artificial intelligence-based hierarchical framework for drug–drug interaction prediction using Morgan fingerprint-based drug-pair representations. **Methods:** At Level 1, Logistic Regression, Random Forest, Linear SVM, and XGBoost were compared for pharmacokinetic/pharmacodynamic (PK/PD) classification. At Level 2, an XGBoost multiclass classifier was used to predict 61 interaction subtype classes. End-to-end performance was evaluated using predicted Level 1 routing, and additional drug-identity-disjoint evaluations were conducted to assess generalization when one or both drugs were unseen during training. Y-randomization analyses were performed for both classification levels. **Results:** Linear SVM achieved the best Level 1 performance, yielding an accuracy of 0.8661, a ROC-AUC of 0.9380, and an MCC of 0.7303 on the independent test set. At Level 2, the XGBoost classifier achieved an accuracy of 0.8712, a balanced accuracy of 0.9087, a macro F1-score of 0.9115, and a Top-3 accuracy of 0.9868. Because the Level 2 test partition was also used for algorithm comparison and model selection, these results should be regarded as exploratory and potentially optimistic rather than as an independent final evaluation. When evaluated end-to-end using predicted Level 1 routing, performance decreased to an accuracy of 0.5767, balanced accuracy of 0.3573, and macro F1-score of 0.4379. Additional drug-identity-disjoint evaluations showed further performance reductions when one or both drugs were unseen during training, highlighting the greater difficulty of generalization to previously unseen drug identities. Y-randomization analyses supported the robustness of both classification levels. **Conclusions:** The framework was deployed as a publicly accessible web platform integrating documented interaction lookup, hierarchical PK/PD classification, Level 1-constrained subtype prediction, confidence scoring, single-pair and batch analysis, ranked Top-3 predictions, and downloadable reports. These findings indicate that upstream routing and unseen-drug generalization remain important limitations of the current framework. DDI-HierPred therefore provides a computational platform for research-oriented screening, interpretation, and prioritization of potential drug–drug interactions.

## 1. Introduction

Polypharmacy, defined as the concurrent use of multiple medications, is increasingly common, particularly among patients with chronic diseases such as diabetes, hypertension, and heart failure [1]. Although combined drug therapy is often clinically necessary, it increases the risk of drug–drug interactions (DDIs), which may reduce therapeutic efficacy or enhance toxicity and adverse effects. DDIs are generally classified into pharmacokinetic and pharmacodynamic interactions [2]. Pharmacokinetic interactions alter drug absorption, distribution, metabolism, or excretion and may consequently modify plasma concentration and duration of action. Pharmacodynamic interactions occur when one drug modifies the effect of another at the receptor, pathway, or physiological level, producing synergistic, additive, or antagonistic effects [3].

PK/PD modeling is increasingly important in precision medicine because it helps relate drug exposure to therapeutic response and toxicity across patients with different clinical and biological profiles. In the context of polypharmacy, PK/PD-oriented modeling can support the interpretation of interaction mechanisms and help prioritize drug combinations that may require closer evaluation. Artificial intelligence and machine-learning approaches further enhance this process by learning complex patterns from documented interaction data and providing decision-support tools for the early identification of potentially relevant PK or PD drug–drug interactions.

The experimental and clinical evaluation of all possible drug combinations is costly and time-consuming because of the rapidly increasing number of potential drug pairs [4]. Machine-learning approaches provide an efficient alternative by learning patterns from documented interactions and using molecular and pharmacological information to predict previously unreported DDIs. Depending on the developed approach, predictive features may include chemical structures, enzymes, molecular targets, adverse-effect profiles, therapeutic classifications, and biological pathways. These computational methods can support the prioritization of potentially risky drug combinations for further experimental or clinical investigation [5,6].

Several machine-learning and deep-learning models have been proposed for DDI prediction. DeepDDI introduced the prediction of multiple interaction types from drug structural information [7], whereas Decagon employed graph neural networks to predict polypharmacy-related adverse effects [8]. DDIMDL subsequently integrated chemical structures with information on enzymes, targets, and biological pathways [9]. More recent approaches, including AutoDDI and RareDDIE [10], have explored advanced neural architectures and the prediction of rare interactions with limited training examples. Despite these advances, many existing methods either predict the presence of an interaction or directly assign a detailed interaction type in a single step. Moreover, predictive models are not always integrated with a practical interface that combines the retrieval of documented interactions with model-based prediction for unlisted drug pairs [11,12].

To address these limitations, the present study developed a hierarchical machine-learning framework for DDI prediction. The proposed workflow comprises two successive classification levels: Level 1 distinguishes pharmacokinetic from pharmacodynamic interactions, while Level 2 predicts a detailed interaction subtype. Drug pairs were represented using concatenated Morgan molecular fingerprints generated from the chemical structures of the interacting drugs. Several machine-learning algorithms were evaluated at both classification levels. Linear SVM was selected for Level 1, whereas XGBoost was selected as the final classifier for Level 2 based on comparative performance. The complete workflow was implemented in the DDI-HierPred web platform, which integrates retrieval of documented interactions with predictive modeling for previously unlisted drug pairs. The platform supports single-pair analysis, batch screening, confidence score reporting, Top-3 subtype prediction, and downloadable results. This framework therefore provides an accessible and research-oriented computational tool for systematic DDI screening and prioritization.

## 2. Materials and Methods

The dataset used in this study was constructed from publicly available DeepDDI/DrugBank-derived resources, which provided documented drug–drug interaction records, DrugBank identifiers, molecular structures, and interaction type descriptions [13]. The principal source files used for dataset construction included DrugBank_known_ddi.txt, Drug_info_combined.csv, Interaction_information.txt, and Approved_drug_enzyme_Information.txt. The initial DDI collection contained 1,420,072 interaction records. Of these, 1,355,205 records were assigned to PK or PD classes for Level 1 dataset construction. Independently, assessment of structural coverage identified 996,112 interaction records for which valid SMILES representations were available for both drugs. For Level 2 subtype modeling, subtype preprocessing, class filtering, and canonicalization resulted in 220,425 unique canonical drug pairs. These counts correspond to distinct preprocessing and task-specific dataset stages and should therefore not be interpreted as successive reductions of a single dataset (Table 1).

**Figure 1:**
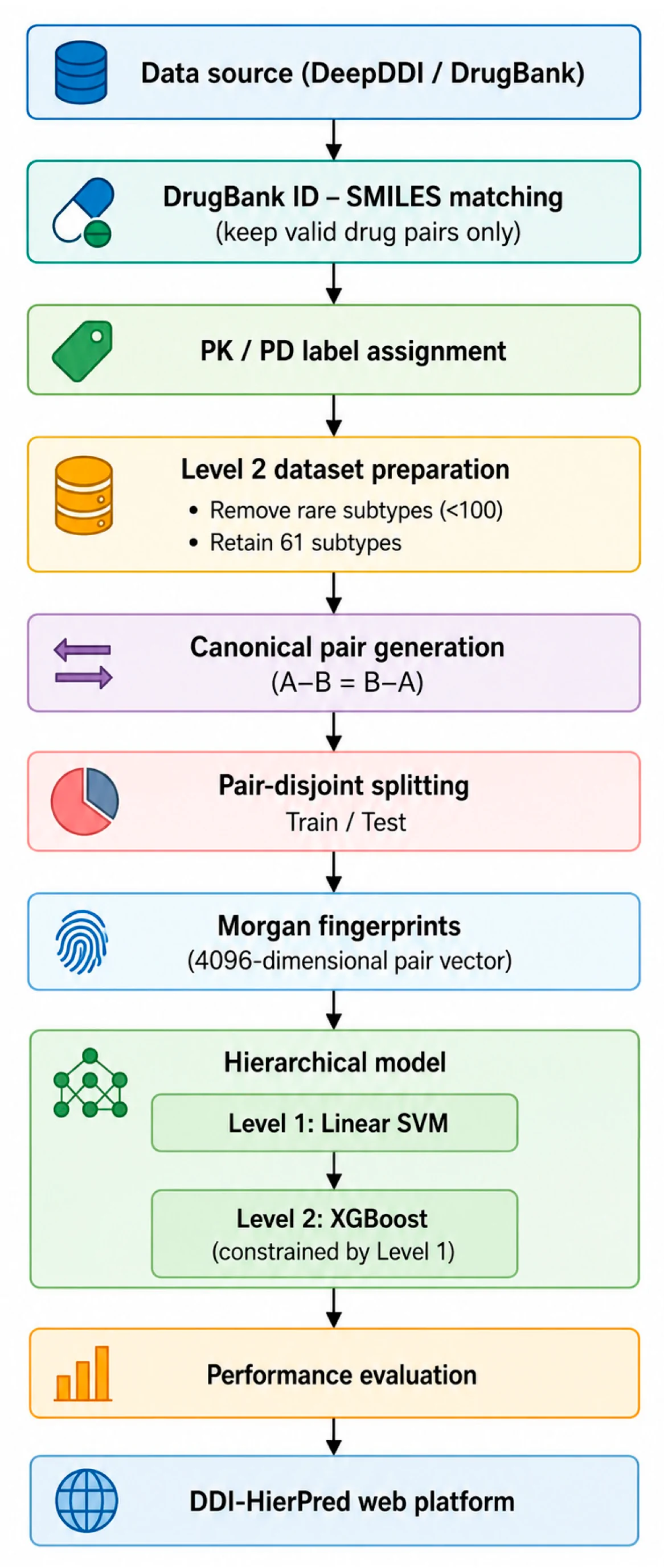
Dataset construction and hierarchical model development workflow of DDI-HierPred.

In addition, a documented DDI lookup table was prepared from the curated interaction resource and integrated into the web platform to retrieve available reference interactions separately from model-generated predictions. The complete dataset construction pipeline, including record filtering, subtype refinement, canonical pair generation, pair-disjoint splitting, and final dataset allocation, is summarized in Figure 1. Detailed file-level documentation and reproducibility materials are provided in the accompanying Zenodo repository.

### 2.1. Construction of Level 1 PK/PD Labels

To construct the first level of the hierarchical classification framework, the original drug–drug interaction descriptions were converted into a binary mechanistic classification scheme. Each interaction subtype was manually reviewed and assigned to either a pharmacokinetic (PK) or pharmacodynamic (PD) category.

Pharmacokinetic interactions were defined as interactions that alter drug exposure through changes in absorption, distribution, metabolism, or excretion. Accordingly, interaction descriptions referring to altered metabolism, changes in serum concentration, clearance, transport, or elimination were assigned to the PK class [14].

Pharmacodynamic interactions were defined as interactions that modify the pharmacological or physiological response of a drug without necessarily affecting its concentration. Descriptions related to increased toxicity, enhanced or reduced therapeutic effects, additive adverse reactions, QT prolongation, bleeding risk, hypotensive effects, sedative effects, or other clinically relevant response changes were assigned to the PD class [15].

Following this mapping process, each interaction subtype was linked to a corresponding Level 1 label (PK or PD), which was subsequently assigned to all drug pairs in the curated dataset. The resulting binary labels were used to train the Level 1 classifier, whereas the original interaction subtypes were retained for Level 2 prediction within the hierarchical framework.

### 2.2. Feature Representation and Drug-Pair Encoding

Each drug–drug interaction was represented as a paired molecular input composed of the structural features of the two interacting drugs. Following the dataset construction workflow described in Figure 1, only drug pairs with valid SMILES representations and curated interaction labels were retained for model development after matching DrugBank identifiers with their corresponding molecular structures.

Molecular structures were encoded from SMILES strings using circular Morgan fingerprints (radius = 2, 2048 bits), as implemented in RDKit [16]. A 2048-bit fingerprint was generated for each drug, and the fingerprints of the two drugs were concatenated to produce a fixed-length 4096-dimensional feature vector representing each drug–drug interaction pair.

This Morgan fingerprint-based representation was used as the common input feature matrix for both levels of the hierarchical prediction framework [17]. In Level 1, the encoded drug pairs were used to classify interactions as pharmacokinetic (PK) or pharmacodynamic (PD). In Level 2, the same molecular pair representation served as the input for multiclass prediction of the retained 61 interaction subtype classes.

For drug pairs associated with more than one documented interaction subtype, each pair–subtype association was retained as a separate single-label record. All records associated with the same canonical drug pair were grouped before partitioning and assigned to the same data split.

### 2.3. Model Development and Training

The Level 1 classification model was developed to distinguish pharmacokinetic from pharmacodynamic drug–drug interactions using Morgan fingerprint-based drug-pair representations. Each drug pair was represented by a 4096-dimensional feature vector obtained by concatenating the 2048-bit Morgan fingerprints of the two interacting drugs and was associated with a binary PK/PD label.

A reproducible stratified sample of 100,000 unique canonical drug pairs was selected for Level 1 model development. Drug-pair order was canonicalized before partitioning so that reversed representations of the same pair were treated as a single pair. The resulting dataset was divided into three mutually exclusive subsets: 80,000 pairs (80%) for training, 10,000 pairs (10%) for internal validation, and 10,000 pairs (10%) for final testing, while preserving the PK/PD class distribution (random_state = 42). The training set was used for model fitting, the validation set for algorithm comparison and model selection, and the final test set was reserved exclusively for evaluation of the selected Level 1 model.

Four machine-learning algorithms were evaluated for Level 1 classification using the same Morgan fingerprint representation: Logistic Regression, Random Forest, Linear SVM, and XGBoost. Linear SVM achieved the best overall validation performance and was therefore selected as the final Level 1 classifier. The selected model was trained with C = 1.0, tol = 0.001, max_iter = 3000, and random_state = 42.

During training, the model learned structural patterns associated with PK and PD interaction classes directly from the concatenated Morgan fingerprint representations of drug pairs.

### 2.4. Software and Computational Environment

All computational analyses were performed using Python 3.10. Data manipulation and numerical operations were carried out using pandas 2.2.2 and NumPy 1.26.4. Molecular structures were processed with RDKit [16], and Morgan fingerprints were generated from SMILES representations. Dataset splitting, label encoding, and model evaluation were performed using scikit-learn 1.3.0 [18]. The trained models were serialized using joblib 1.2.0, while the web-based prediction interface was developed using Gradio 5.27.0 [19].

### 2.5. Model Evaluation Metrics

The predictive performance of the Level 1 classification models was evaluated on the test set using metrics implemented in scikit-learn 1.3.0 [18]. Accuracy, precision, recall, F1-score, ROC-AUC, Matthews correlation coefficient (MCC), and the confusion matrix were calculated to assess overall performance, class-specific predictive ability, and discrimination between pharmacokinetic and pharmacodynamic drug–drug interactions.

Accuracy represented the proportion of correctly classified drug pairs, whereas precision and recall measured the reliability and sensitivity of class predictions. The F1-score provided a balanced measure of precision and recall, while ROC-AUC assessed the ability of the model to discriminate between PK and PD classes across different classification thresholds.

MCC was additionally included because it provides a robust summary of binary classification performance, particularly in the presence of class imbalance. A confusion matrix was also generated to summarize correct and incorrect predictions for each class.

### 2.6. End-to-End Hierarchical Evaluation

To evaluate the complete hierarchical workflow, Level 2 subtype prediction was assessed under three configurations: (i) the conventional flat 61-class Level 2 classifier without PK/PD branch restriction; (ii) oracle hierarchical routing, in which Level 2 predictions were restricted using the true Level 1 PK/PD labels; and (iii) predicted hierarchical routing, in which the PK/PD branch predicted by the Level 1 Linear SVM was used to constrain Level 2 predictions. To ensure a direct comparison, all three configurations were evaluated on the same 43,975 Level 2 test records for which exact Level 1 predictions could be generated, corresponding to 99.75% of the 44,086-record pair-disjoint Level 2 test set.

As shown in Table 2, oracle hierarchical routing produced the highest overall performance, achieving an accuracy of 0.9243, balanced accuracy of 0.9374, macro F1-score of 0.9378, MCC of 0.9127, and Top-3 accuracy of 0.9935. These values exceeded those of the unrestricted flat 61-class classifier, which achieved an accuracy of 0.8712, balanced accuracy of 0.9086, macro F1-score of 0.9114, MCC of 0.8507, and Top-3 accuracy of 0.9867. Thus, when the correct PK/PD branch was available, hierarchical restriction improved subtype classification by eliminating predictions belonging to the incompatible mechanistic branch.

In contrast, when routing was based on the actual Level 1 predictions, end-to-end performance decreased to an accuracy of 0.5767, balanced accuracy of 0.3573, macro F1-score of 0.4379, MCC of 0.4968, and Top-3 accuracy of 0.6230. The Level 1 routing accuracy on this matched Level 2 evaluation subset was 0.6264. The difference between oracle and predicted routing demonstrates that errors introduced at Level 1 propagate directly to downstream subtype classification. Accordingly, although branch-constrained prediction improves Level 2 classification when the upstream PK/PD assignment is correct, the end-to-end results identify Level 1 generalization as a principal limitation of the current hierarchical pipeline. The lower Level 1 accuracy observed on the matched Level 2 evaluation subset (0.6264) compared with the primary Level 1 test set (0.8661) should be interpreted in the context of the different evaluation populations. The primary Level 1 test set contained 45.65% PK and 54.35% PD interactions, whereas the matched Level 2 subset was substantially enriched in PK interactions (69.83% PK and 30.17% PD). The performance reduction was predominantly associated with the PK class, for which recall decreased from 0.8561 to 0.5445, while PD recall decreased more modestly from 0.8745 to 0.8159. Drug coverage alone did not explain this difference: 97.63% of the matched Level 2 pairs contained two drugs already represented in the Level 1 training set, yet accuracy within this group was 0.6296. These findings indicate that the lower performance primarily reflects differences in the composition and class distribution of the Level 2 evaluation subset, particularly its greater representation of PK interactions, rather than being driven mainly by previously unseen drugs.

### 2.7. Level 2 Interaction Subtype Model

The original DeepDDI resource contained 113 interaction subtype classes. Following dataset curation, 52 underrepresented subtypes were excluded, resulting in a final set of 61 interaction subtype classes used for Level 2 model development (Table 3). Interaction subtypes represented by fewer than 100 samples were removed to improve the stability and reliability of multiclass classification. The complete mapping between the original 113 interaction subtype classes and the final 61 retained classes, together with the corresponding dataset audit tables, is available in the Zenodo repository (see Data Availability Statement).

After excluding interaction subtypes represented by fewer than 100 samples, the retained records were used to construct the final Level 2 dataset without imposing a fixed maximum number of samples per class. The resulting dataset contained 220,425 interaction records across 61 subtype classes. Because the retained subtype distribution remained imbalanced, model performance was assessed using class-sensitive metrics, including balanced accuracy, macro precision, macro recall, macro F1-score, MCC, and a majority-class baseline. After feature generation and removal of drug pairs without complete molecular representations, the Level 2 feature matrix was constructed using the same 4096-dimensional Morgan fingerprint-based drug-pair representation employed in the hierarchical framework.

To ensure strict pair-disjoint evaluation, drug pairs were first converted into a canonical representation by sorting the two DrugBank identifiers alphabetically, making (Drug A, Drug B) and (Drug B, Drug A) equivalent. The dataset was then partitioned at the canonical pair level so that all records belonging to the same drug pair remained within a single partition. Consequently, duplicate annotations, reversed drug pairs, and repeated interaction records were prevented from being distributed across both the training and test sets. Post-splitting verification confirmed that no canonical drug pair was shared between the two partitions (pair leakage = 0).

Logistic Regression (SGD implementation), Random Forest, and XGBoost classifiers were evaluated using the same pair-disjoint training and test partitions. Based on the overall multiclass performance, XGBoost was selected as the final Level 2 classifier.

The final multiclass XGBoost model was trained using the multi:softprob objective function with n_estimators = 300, max_depth = 6, learning_rate = 0.05, subsample = 0.8, colsample_bytree = 0.8, eval_metric = mlogloss, tree_method = hist, random_state = 42, and n_jobs = −1.

Model performance was evaluated using multiclass classification metrics, including accuracy, balanced accuracy, macro precision, macro recall, macro F1-score, weighted F1-score, Matthews correlation coefficient (MCC), multiclass log loss, and Top-3 accuracy. In addition, a majority-class baseline model was evaluated for comparison, and model robustness was further assessed using Y-randomization with 20 independent label permutations [15]. Additional drug-identity-disjoint evaluation protocols were implemented to quantify generalization beyond previously observed drug identities. In addition to the canonical pair-disjoint split, two more stringent settings were considered. For each repeated split, 20% of the unique drugs (369 drugs) were randomly selected without replacement as held-out drugs. Drug pairs containing no held-out drugs were assigned to the training set; pairs containing exactly one held-out drug constituted the one-unseen-drug test set; and pairs in which both drugs were held out constituted the fully drug-disjoint test set. Candidate splits were generated using random seeds and screened to ensure that all 61 retained Level 2 subtypes remained represented in the training partition. The first five splits satisfying this criterion, corresponding to random seeds 10, 11, 12, 14, and 17, were retained for evaluation. Across these five splits, the mean proportions of records assigned to the training, one-unseen-drug, and fully drug-disjoint partitions were 64.19%, 31.94%, and 3.94%, respectively. All 61 subtypes were represented in the training and one-unseen-drug partitions of each retained split, whereas the fully drug-disjoint partitions contained 49–54 subtypes because no requirement for complete subtype coverage was imposed on this more restrictive test setting. Performance variability was summarized as mean ± standard deviation across the five splits. Drug-identity overlap between the conventional pair-disjoint training and test partitions was also explicitly quantified.

### 2.8. Prediction Scores and Confidence Reporting

For the Level 1 Linear SVM classifier, the numerical confidence score displayed by DDI-HierPred was derived from the model decision function. The signed decision-function output was transformed to the [0, 1] interval using a sigmoid function, s = 1/(1 + e^−d^), where d denotes the Linear SVM decision score. The complementary scores were assigned to the PK and PD classes according to the model class ordering, and the class with the higher score was reported as the Level 1 prediction. Because no probability calibration procedure was applied, these values are reported strictly as confidence scores and should not be interpreted as calibrated class probabilities.

For Level 2, XGBoost class scores obtained from predict_proba were used to rank interaction subtypes. Following the hierarchical constraint, only subtypes belonging to the Level 1-predicted PK or PD branch were eligible for Top-1 and Top-3 reporting. The original Level 2 model scores were retained without renormalization after exclusion of branch-incompatible subtypes. Accordingly, the platform reports these numerical outputs as confidence scores rather than calibrated probabilities.

### 2.9. Y-Randomization Validation

To assess whether the observed predictive performances were attributable to meaningful structure–interaction relationships rather than chance correlations, Y-randomization analyses were performed for both classification levels [20]. For Level 1, the PK/PD labels were randomly shuffled after dataset partitioning, and the selected Linear SVM model was retrained 20 times using the same Morgan fingerprint-based representation and training procedure. For Level 2, the interaction subtype labels were randomly shuffled after the pair-disjoint dataset partitioning, and the XGBoost classifier was retrained 20 times using the same feature representation, model settings, and training protocol. The performances of the randomized models were then compared with those of the corresponding original models using the same evaluation metrics.

### 2.10. Web Interface Development

The proposed hierarchical prediction framework was implemented as a web-based application developed in Python using Gradio 5.27.0 [19]. The application integrated the cleaned interaction resource, curated drug information, trained machine-learning models, and the feature-generation pipeline within a unified computational environment. It was subsequently deployed on Hugging Face Spaces (https://huggingface.co/) [21], enabling browser-based access without requiring local installation.

## 3. Results

### 3.1. Curated Dataset and Feature Matrix

After data cleaning and molecular structure validation, drug pairs with available SMILES representations were retained for model development. Each drug–drug interaction pair was represented using concatenated Morgan fingerprints, generating a 4096-dimensional feature vector. The complete cleaned PK/PD interaction resource containing 1,355,205 documented drug–drug interaction pairs was retained separately for the known-interaction lookup module integrated into the DDI-HierPred platform.

### 3.2. Level 1 PK/PD Model Performance

A reproducible stratified sample of 100,000 unique canonical drug pairs was used for Level 1 model development and partitioned into training (80,000 pairs; 80%), internal validation (10,000 pairs; 10%), and final test (10,000 pairs; 10%) subsets while preserving the PK/PD class distribution. The internal validation set was used for algorithm comparison and model selection, whereas the final test set was reserved exclusively for evaluation of the selected model. Four machine-learning algorithms were evaluated using the same Morgan fingerprint-based drug-pair representation: Logistic Regression, Random Forest, Linear SVM, and XGBoost.

**Figure 2:**
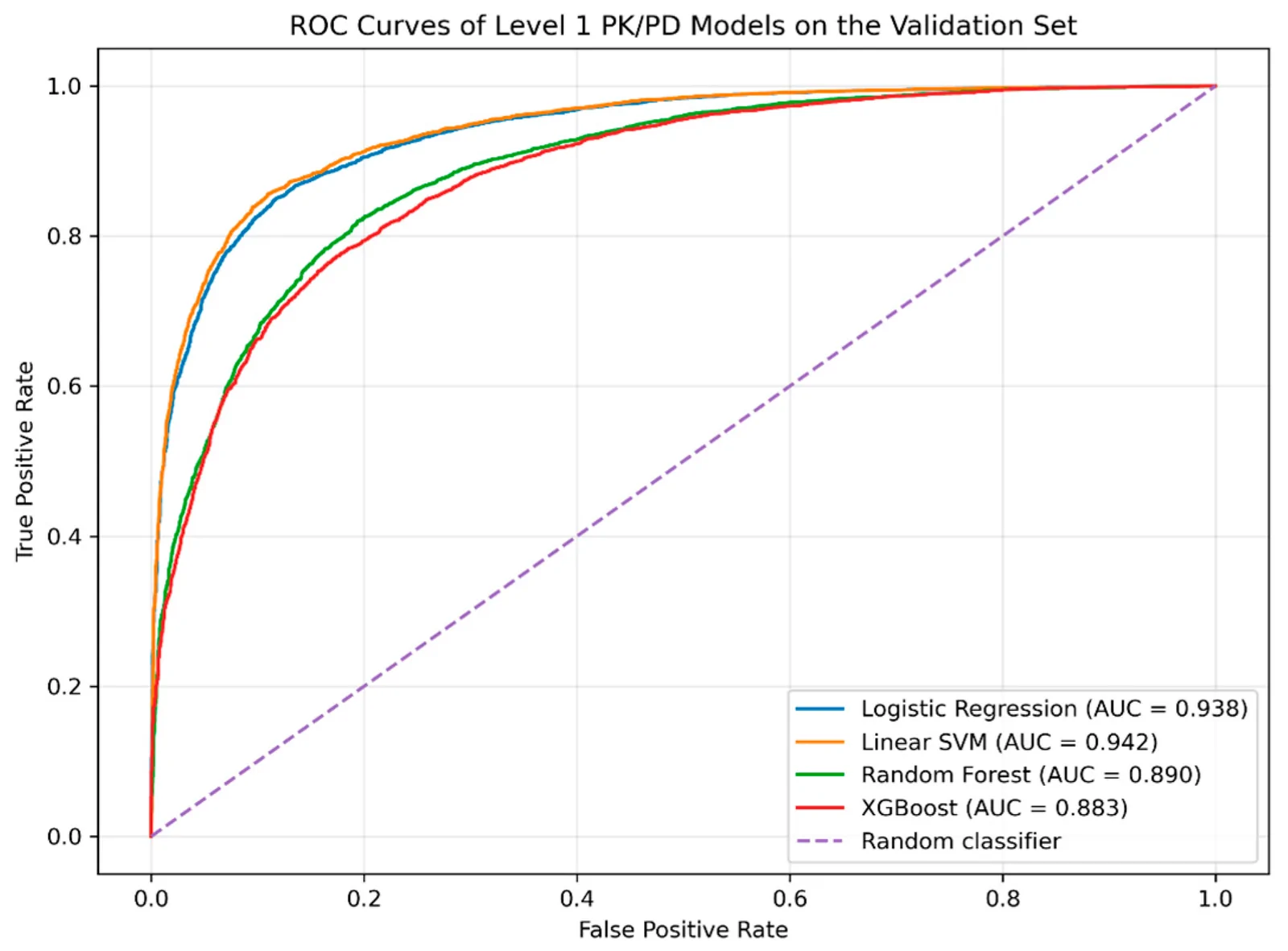
ROC curves of Level 1 PK/PD models on the validation set.

As shown in Table 4 and Figure 2, Linear SVM achieved the best overall performance on the internal validation set, with an accuracy of 0.8683, a ROC-AUC of 0.9418, and an MCC of 0.7347. Logistic Regression showed comparable performance, reaching a ROC-AUC of 0.9383, whereas Random Forest and XGBoost achieved lower ROC-AUC values of 0.8904 and 0.8827, respectively. Based on these results, Linear SVM was selected as the final Level 1 classifier.

The selected Linear SVM model was subsequently evaluated on the independent final test set (Table 5), achieving an accuracy of 0.8661, a ROC-AUC of 0.9380, and an MCC of 0.7303. The confusion matrix showed that the model correctly classified 3908 PK interactions and 4753 PD interactions, while 657 PK interactions were misclassified as PD and 682 PD interactions were misclassified as PK. The similar performance observed on the internal validation and independent test sets indicates stable generalization without evidence of substantial performance degradation.

### 3.3. Robustness Assessment by Y-Randomization

To evaluate whether the predictive performance of the selected Linear SVM model resulted from genuine structure–activity relationships rather than chance correlations, a Y-randomization test was performed [20]. The PK/PD labels were randomly shuffled, and the model was retrained 20 times using the same feature representation and training procedure.

**Figure 3:**
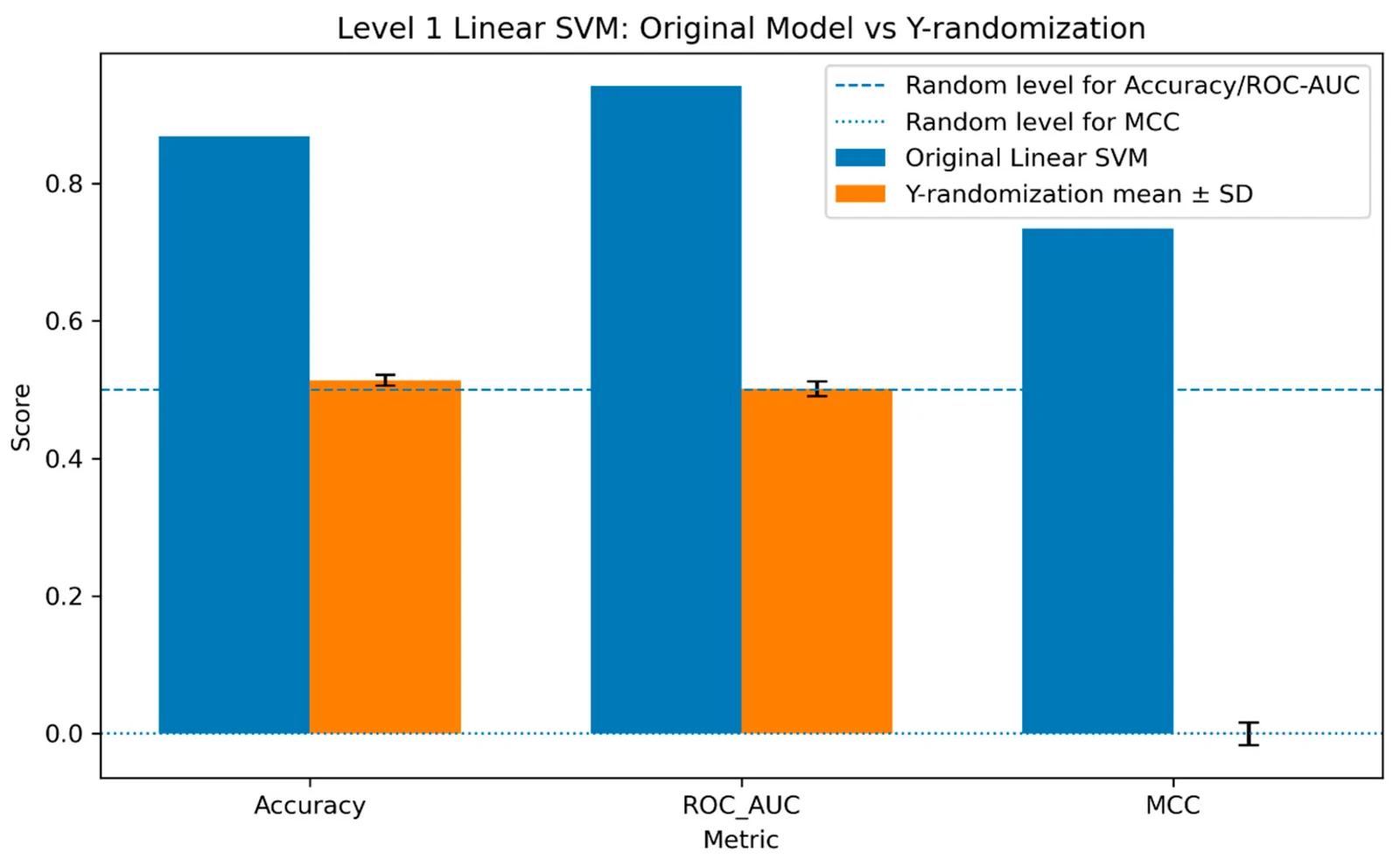
Y-randomization validation of the Level 1 PK/PD classifier.

As shown in Figure 3, the original Linear SVM model substantially outperformed the randomized models across all evaluation metrics. The original model achieved an accuracy of 0.8683, a ROC-AUC of 0.9418, and an MCC of 0.7347, whereas the randomized models produced average ROC-AUC and accuracy values close to random classification and MCC values near zero (Table 6).

The large performance gap between the original and randomized models indicates that the predictive ability of the proposed model originates from meaningful relationships captured within the molecular representations rather than from random correlations in the dataset. These results confirm the robustness and reliability of the Level 1 PK/PD classification model.

### 3.4. Level 2 Interaction Subtype Classification

The original interaction resource contained 113 documented interaction subtypes. After excluding 52 underrepresented interaction categories, 61 interaction subtype classes were retained for multiclass model development. To prevent direct pair-level information leakage, canonical drug pairs were generated such that A–B and B–A were treated as the same pair, and the dataset was partitioned using a pair-disjoint splitting strategy. Consequently, all records associated with a given canonical drug pair were assigned exclusively to either the training or the test partition.

An audit of the resulting dataset confirmed that no canonical drug pair was shared between the training and test sets (pair leakage = 0%). As summarized in Table 7, the dataset contained 297 repeated ordered pairs, 196 reversed drug pairs, and 423 canonical drug pairs associated with more than one interaction subtype annotation. All records belonging to the same canonical pair were retained within a single partition, thereby preventing repeated or reversed representations of the same drug pair from crossing the training–test boundary.

Further inspection of the 423 multi-annotated canonical pairs showed that 363 were associated with multiple subtypes belonging to the same PK/PD branch, whereas 60 contained subtype annotations spanning both PK and PD branches. Overall, these multi-annotated pairs represented only 0.1923% of the unique Level 2 drug pairs. Because the Level 2 classifier was formulated as a single-label multiclass model, each documented pair–subtype association was retained as a separate labeled record rather than being encoded as a multilabel target. Consequently, a drug pair associated with multiple documented subtypes could contribute more than one record, with each record carrying one subtype label. Canonical pair grouping was performed before data partitioning, ensuring that all records and subtype annotations associated with the same drug pair were assigned exclusively to the same partition and could not cross partition boundaries. Accordingly, the present Level 2 formulation should be interpreted as classification of documented pair–subtype associations rather than as a formal multilabel prediction framework. A dedicated multilabel formulation capable of jointly predicting multiple interaction subtypes for the same drug pair was not implemented in the present study.

### 3.5. Drug-Identity-Disjoint Generalization Assessment

Although the canonical pair-disjoint split completely eliminated overlap of canonical drug pairs between the training and test sets, substantial overlap remained at the individual-drug level. Specifically, the training and test partitions contained 1828 and 1732 unique drugs, respectively, of which 1716 were shared between the two partitions. Thus, 99.08% of the drugs represented in the test set had also occurred in the training set, despite zero canonical-pair overlap. This observation motivated the additional one-unseen-drug and fully drug-disjoint evaluations described below.

Although canonical pair-disjoint partitioning prevents the same drug combination from appearing in both training and test sets, it does not necessarily prevent individual drug identities from occurring in both partitions. Drug overlap was therefore quantified, and additional evaluations were conducted under progressively more stringent generalization settings. In the one-unseen-drug setting, each evaluated test pair contained one drug that was absent from the corresponding training set. In the fully drug-disjoint setting, both drugs forming each evaluated test pair were absent from the training set.

The five retained drug-holdout splits used random seeds 10, 11, 12, 14, and 17, with 369 drugs (20% of the unique drug set) held out in each repetition. Across the five splits, the mean proportions of interaction records assigned to the training, one-unseen-drug, and fully drug-disjoint partitions were 64.19%, 31.94%, and 3.94%, respectively. All 61 Level 2 subtypes were represented in the training and one-unseen-drug partitions, whereas 49–54 subtypes were represented in the fully drug-disjoint partitions. Exact sample sizes, numbers of unique drugs, and subtype coverage for each split are provided in Supplementary Table S1.

As summarized in Table 8, predictive performance decreased progressively as the evaluation setting became more stringent. Under the one-unseen-drug protocol, the model achieved a mean accuracy of 0.6578 ± 0.0196, balanced accuracy of 0.6360 ± 0.0284, macro F1-score of 0.6825 ± 0.0269, MCC of 0.5977 ± 0.0208, and Top-3 accuracy of 0.8722 ± 0.0079 across five repeated splits. Under the more stringent fully drug-disjoint protocol, in which neither drug in the evaluated pair had been observed during training, performance decreased to an accuracy of 0.4559 ± 0.0288, balanced accuracy of 0.3387 ± 0.0543, macro F1-score of 0.3717 ± 0.0655, MCC of 0.3380 ± 0.0268, and Top-3 accuracy of 0.7349 ± 0.0135.

The progressive reduction in performance indicates that prediction becomes substantially more challenging as drug-identity overlap with the training data is removed. Nevertheless, performance remained above the corresponding chance-level expectation, particularly for Top-3 prediction, even under the fully drug-disjoint setting. These results distinguish generalization to unseen drug combinations from the substantially more difficult extrapolation to previously unseen drug identities.

### 3.6. Comparison of Level 2 Classification Algorithms

Three machine-learning algorithms—XGBoost, Random Forest, and SGD Logistic Regression—were evaluated using the same Morgan fingerprint-based drug-pair representation and the same pair-disjoint evaluation framework. Their predictive performances are summarized in Table 9.

Random Forest achieved the highest overall accuracy (0.8823), MCC (0.8635), and Top-3 accuracy (0.9900). In contrast, XGBoost achieved substantially higher balanced accuracy (0.9087) and macro F1-score (0.9115), indicating more consistent predictive performance across the 61 interaction subtype classes. Random Forest yielded a balanced accuracy of 0.8406 and a macro F1-score of 0.8740, while SGD Logistic Regression achieved 0.7975 and 0.8301, respectively. Because the Level 2 dataset is strongly imbalanced across interaction subtypes, balanced accuracy and macro-averaged performance were prioritized over overall accuracy alone. XGBoost was therefore selected as the final Level 2 classifier.

The selected XGBoost model also achieved a Top-3 accuracy of 0.9868, indicating that the documented interaction subtype was included among the three highest-ranked predictions for most evaluated records.

To provide a simple reference for model performance under class imbalance, a majority-class baseline was additionally evaluated. The baseline achieved an accuracy of 0.2865 but a balanced accuracy of only 0.0164, a macro F1-score of 0.0073, and an MCC of 0.0000. These results illustrate the inadequacy of overall accuracy alone for evaluating this highly imbalanced multiclass problem and support the use of balanced and macro-averaged metrics for model comparison.

**Figure 4:**
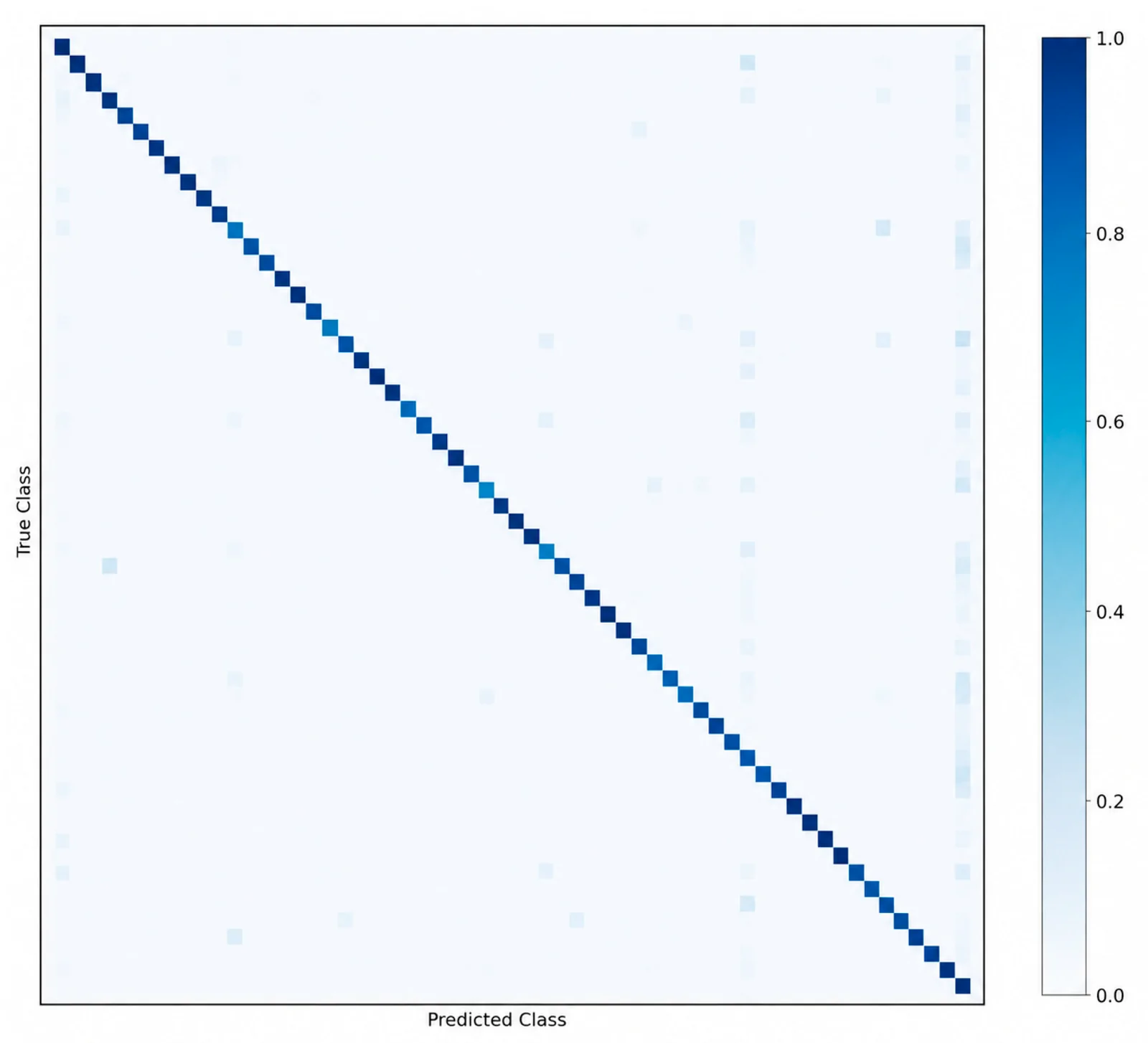
Normalized confusion matrix of the selected XGBoost model on the pair-disjoint test set. Darker diagonal cells indicate correct classification, whereas off-diagonal cells represent misclassified interaction subtypes.

The class-wise predictive performance of the selected XGBoost model is illustrated by the normalized confusion matrix (Figure 4). Most interaction subtypes show high values along the main diagonal, indicating correct classification for a substantial proportion of samples within their respective classes. Off-diagonal predictions are concentrated in a more limited subset of class combinations, indicating that misclassification is not uniformly distributed across all 61 subtypes. Together with the balanced accuracy and macro F1-score, the confusion matrix supports the ability of the model to discriminate among the retained interaction subtype classes under the pair-disjoint evaluation setting.

### 3.7. Robustness Assessment of the Level 2 Model by Y-Randomization

To evaluate whether the predictive performance of the selected Level 2 XGBoost model resulted from meaningful structure–interaction relationships rather than chance correlations, a Y-randomization test was performed. The interaction subtype labels were randomly shuffled after the pair-disjoint train/test partition had been established while preserving the original feature representation, training procedure, and model hyperparameters. The randomized models were retrained 20 times under identical experimental conditions and evaluated on the unchanged independent test set.

**Figure 5:**
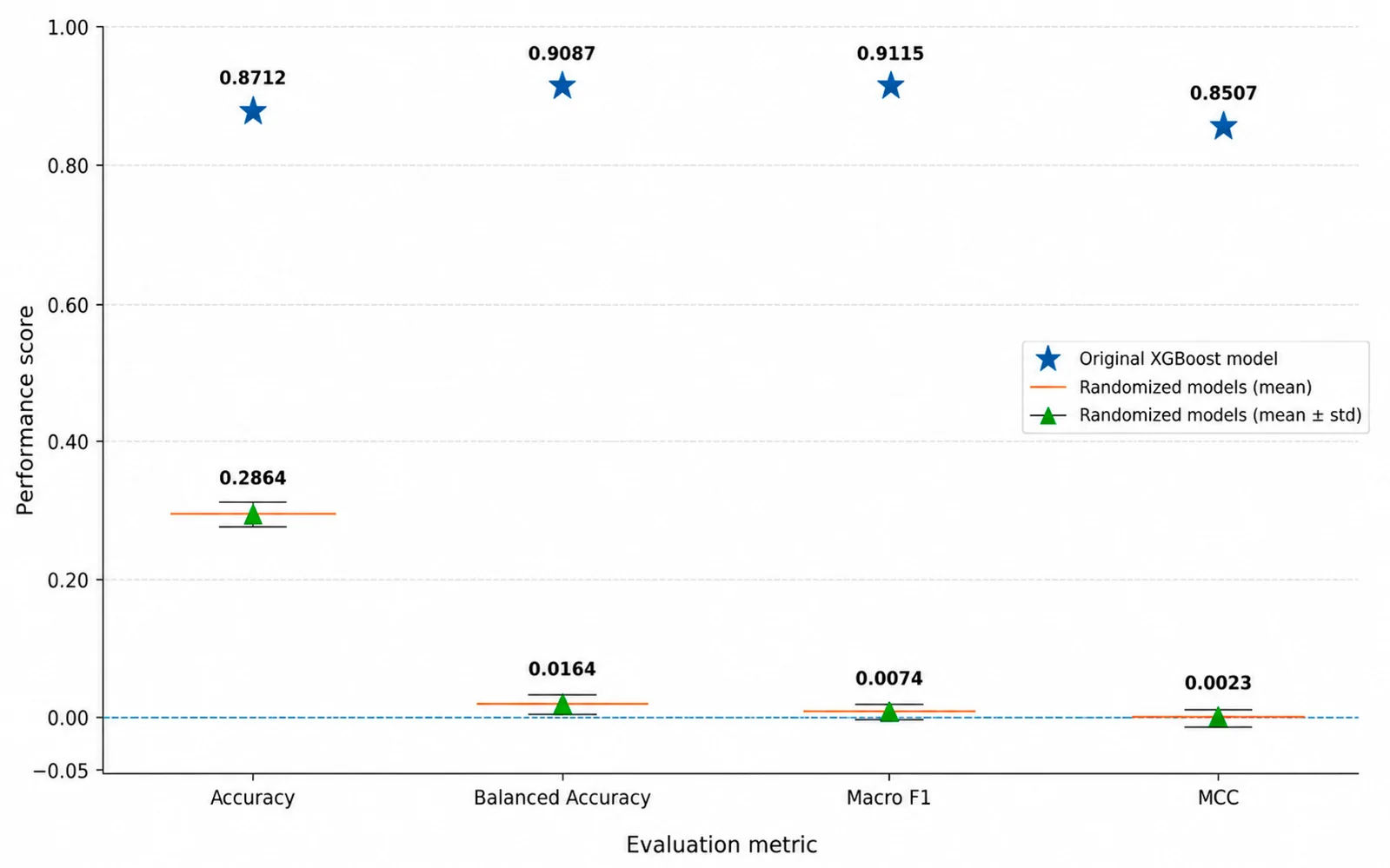
Comparison between the original Level 2 XGBoost model and Y-randomized models.

As shown in Figure 5, the randomized models exhibited dramatically lower predictive performance than the original model. The original XGBoost classifier achieved an accuracy of 0.8712, a balanced accuracy of 0.9087, and a macro F1-score of 0.9115. In contrast, the randomized models produced performance values close to random classification, with mean accuracy, balanced accuracy, and macro F1-score values of 0.2864, 0.0164, and 0.0074, respectively (Table 10).

An empirical significance analysis further confirmed these observations. None of the randomized models achieved performance comparable to that of the original classifier, yielding an empirical *p*-value of 0.0476 across all evaluated performance metrics (20 permutations). These results indicate that the probability of obtaining the observed predictive performance by chance is very low.

Overall, the substantial performance gap between the original and randomized models demonstrates that the predictive capability of the Level 2 classifier originates from meaningful structure–interaction relationships captured by the molecular fingerprint representation rather than from random correlations or data-partitioning artifacts.

### 3.8. Web-Based DDI Prediction Platform

**Figure 6:**
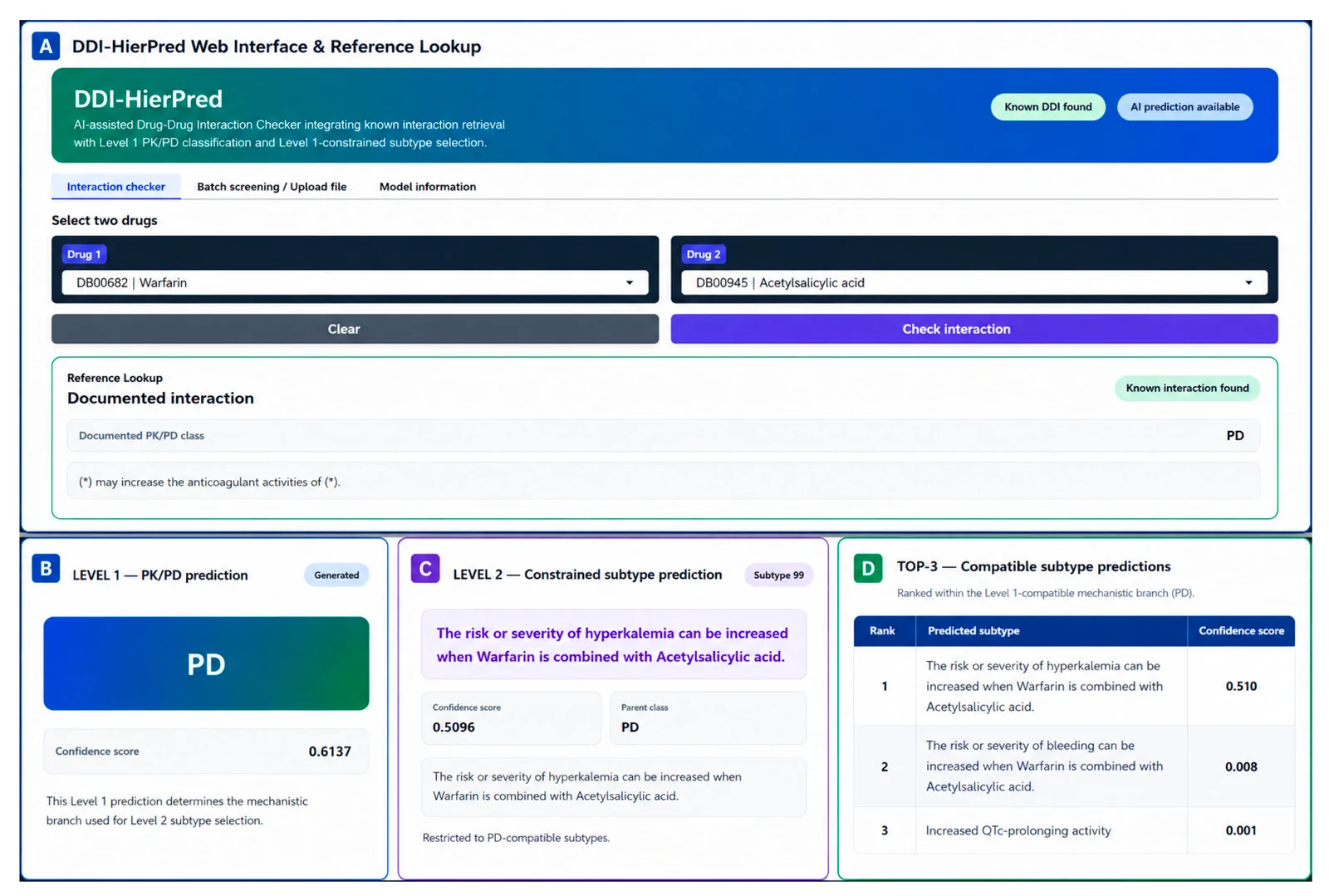
DDI-HierPred web interface and hierarchical prediction workflow for a representative drug pair. (**A**) Reference lookup for the selected drug pair. (**B**) Level 1 PK/PD prediction. (**C**) Level 1-constrained Level 2 subtype prediction. (**D**) Top-3 subtype predictions restricted to the Level 1-selected mechanistic branch. The asterisk (*) shown in the documented interaction text is a placeholder retained from the original interaction description in the reference dataset and does not indicate statistical significance.

The developed DDI-HierPred platform provides a publicly accessible web-based implementation of the proposed hierarchical drug–drug interaction prediction framework (https://huggingface.co/spaces/Prof-ouassaf/DDI-Hierarchical-Predictor, accessed on 16 June 2026). As illustrated in Figure 6, the interface supports two complementary modes of operation: interactive analysis of a single drug pair and batch screening of multiple drug pairs through CSV file upload.

For each submitted drug pair, the platform first queries an integrated database of documented drug–drug interactions and displays any available known interaction together with its corresponding pharmacokinetic (PK) or pharmacodynamic (PD) category. This lookup module serves as a reference resource for documented interactions and operates independently of the machine-learning prediction models.

The hierarchical prediction workflow subsequently applies the Level 1 Linear SVM classifier to assign the drug pair to either the PK or PD interaction class and reports the associated confidence score. The predicted Level 1 class is then used to constrain the Level 2 XGBoost classifier, which predicts the three most probable Level 2 subtype predictions only within the corresponding mechanistic branch. In addition to the highest-ranked subtype, the platform reports the three most probable Level 2 subtype predictions together with their corresponding confidence scores, providing users with alternative mechanistically plausible interaction hypotheses.

In batch mode, the platform automatically processes all submitted drug pairs and generates downloadable summary reports containing the lookup status, Level 1 classification, confidence scores, predicted interaction subtype, and ranked Top-3 Level 2 predictions for each drug pair. By integrating documented interaction retrieval, hierarchical prediction, confidence estimation, constrained subtype prediction, and batch analysis within a single interface, DDI-HierPred provides a research-oriented platform for the rapid and interpretable assessment of documented and previously unlisted drug–drug interactions. Predictions generated for previously unlisted drug pairs should be regarded as computational hypotheses requiring independent experimental or clinical validation.

### 3.9. Hierarchical Branch-Consistency Assessment

To quantitatively assess the hierarchical consistency of the DDI-HierPred framework, Level 2 predictions were evaluated across the complete test set (*n* = 44,086) before and after applying the Level 1-based branch constraint. For each drug pair, the predicted Level 2 subtype was considered branch-consistent when its assigned PK/PD parent class matched the mechanistic branch predicted by Level 1.

As shown in Table 11, the raw unconstrained Level 2 outputs showed a branch-consistency rate of 57.30% for Top-1 predictions. For Top-3 predictions, only 7.13% of test pairs had all three predicted subtypes belonging to the Level 1-selected branch. After applying the hierarchical constraint, branch consistency reached 100% for both Top-1 and Top-3 predictions across all 44,086 test pairs. Thus, the constrained implementation ensures that every Level 2 subtype reported by the hierarchical workflow belongs to the PK or PD branch selected by Level 1.

### 3.10. Agreement with Documented PK/PD Annotation

To assess agreement between Level 1 predictions and available reference annotations, the predicted PK/PD class was compared with the lookup-derived PK/PD label for all unique documented drug pairs represented in the test set. After pair-level deduplication, 33,741 documented pairs were available for this analysis. Level 1 predictions agreed with the documented PK/PD classification for 23,491 pairs, corresponding to an overall agreement rate of 69.62%, whereas 10,250 pairs were discordantly classified.

As shown in Table 12, among documented PD pairs, 14,447 were predicted as PD and 5607 as PK. Among documented PK pairs, 9044 were predicted as PK and 4643 as PD. Thus, the analysis explicitly quantifies concordant and discordant Level 1 classifications rather than assuming agreement between model predictions and lookup-derived annotations.

### 3.11. End-to-End Hierarchical Performance

To quantify the performance of the complete hierarchical workflow, Level 1 classification and the subsequent branch-constrained Level 2 prediction were evaluated jointly (Table 13). The analysis included 43,975 drug pairs, corresponding to 99.75% of the full pair-disjoint test set. Level 1 achieved an accuracy of 62.64% on these pairs. When the complete hierarchical prediction was considered, the end-to-end Top-1 subtype accuracy was 57.67%, while the end-to-end Top-3 accuracy reached 62.30%.

To distinguish errors originating from Level 1 routing from those occurring during subtype classification, Level 2 performance was additionally examined conditional on correct Level 1 classification. Among pairs correctly classified at Level 1, the subtype Top-1 accuracy reached 92.08%, and the Top-3 accuracy reached 99.46%. Of the 43,975 evaluated pairs, 16,431 errors originated at Level 1. Among pairs correctly routed by Level 1, 2182 subsequently missed the correct Top-1 subtype, whereas only 149 missed the correct subtype within the Top-3 predictions. These results indicate that errors in the complete hierarchical pipeline were predominantly associated with Level 1 routing rather than with subtype ranking after correct branch assignment.

### 3.12. Representative Application of the DDI-HierPred Web Platform

To illustrate the operation of the updated DDI-HierPred platform, ten representative drug pairs were evaluated using the deployed hierarchical workflow. For each pair, the platform first performs reference lookup and then independently generates a Level 1 PK/PD prediction followed by Level 2 subtype prediction constrained to the mechanistic branch selected by Level 1. The resulting reference and predictive outputs are summarized in Table 14.

Among the ten evaluated pairs, three were identified in the reference lookup resource as documented interactions, whereas seven were not retrieved from the lookup dataset. The Level 1 model classified five pairs as PK and five as PD, providing representative examples from both mechanistic branches. For the three lookup-positive pairs, the documented interaction and its PK/PD annotation are displayed separately from the model-generated prediction. For the remaining seven pairs, the platform reports only the model-generated hierarchical prediction and explicitly indicates that no interaction was found in the lookup resource.

Importantly, the Level 2 predictions followed the branch selected at Level 1 in all ten representative examples. PK-routed pairs were assigned only PK-compatible Level 2 subtypes, whereas PD-routed pairs were assigned only PD-compatible subtypes. This behavior reflects the branch-restriction mechanism implemented in the revised platform. The representative examples presented in Table 14 are intended to illustrate the practical operation and output structure of the deployed system rather than to constitute an independent performance evaluation. Quantitative branch consistency was assessed separately across the complete test set, as reported in Section 3.9.

## 4. Discussion

The present study introduced DDI-HierPred as a hierarchical machine-learning framework for drug–drug interaction prediction. Unlike several previous approaches that directly predict interaction events or detailed interaction types in a single step [7,8,9,10], the proposed framework separates the prediction task into two complementary levels. Level 1 distinguishes pharmacokinetic (PK) from pharmacodynamic (PD) interactions, whereas Level 2 provides a more detailed interaction subtype constrained by the mechanistic branch selected at Level 1. This hierarchical organization provides a structured interpretation of model outputs by linking detailed interaction subtypes to their broader PK or PD category.

Data preparation represented a critical component of the proposed workflow. Drug identifiers, molecular structures, and interaction annotations were harmonized before model construction, and structural coverage was explicitly assessed during dataset preparation. Such curation is important because machine-learning performance can be strongly affected by the quality, consistency, and completeness of input data [11,12]. Morgan fingerprints were used as the molecular representation to provide uniform structural encoding across drug pairs and to avoid dependence on auxiliary biological annotations with incomplete coverage.

For Level 1 prediction, four machine-learning algorithms were evaluated using the same Morgan fingerprint-based drug-pair representation. Linear SVM achieved the best overall performance among the evaluated classifiers and was therefore selected as the PK/PD classifier. This result is consistent with the suitability of support vector machines for high-dimensional feature spaces [14]. Morgan fingerprints generate sparse, high-dimensional molecular representations, for which linear margin-based classifiers can provide effective discrimination while limiting model complexity.

Y-randomization analysis further supported the robustness of the Level 1 model. High predictive performance alone does not exclude the possibility of chance correlations, particularly in cheminformatics and QSAR-like modeling workflows. Following random permutation of the PK/PD labels, predictive performance decreased markedly, with ROC-AUC approaching random classification and MCC values approaching zero. The substantial difference between the original and randomized models indicates that the observed Level 1 performance was associated with relationships between molecular representation and interaction labels rather than random label structure.

For Level 2 interaction subtype classification, XGBoost, Random Forest, and SGD Logistic Regression were compared using the same Morgan fingerprint-based drug-pair representation. Although Random Forest achieved slightly higher overall accuracy and MCC in the reported comparison, XGBoost showed higher balanced accuracy and macro F1-score while maintaining high Top-3 performance. These metrics were particularly relevant given the multiclass and imbalanced nature of the Level 2 dataset, because they provide greater sensitivity to performance across less represented interaction classes. XGBoost was consequently retained as the Level 2 classifier.

The normalized confusion matrix provided additional information on class-specific Level 2 performance. Most interaction subtypes were concentrated along the main diagonal, whereas off-diagonal errors were restricted to a smaller subset of classes. Nevertheless, the presence of confusion among some interaction subtypes indicates that detailed mechanistic classification remains more challenging than broad PK/PD discrimination and that aggregate performance metrics should be interpreted together with class-wise results.

The robustness of the Level 2 classifier was further examined through Y-randomization. After random permutation of interaction subtype labels, performance decreased substantially across all evaluation metrics. The randomized models achieved an average macro F1-score of 0.0074, balanced accuracy of 0.0164, and MCC close to zero, compared with 0.9115, 0.9087, and 0.8507, respectively, for the corresponding original model. This large separation between the original and randomized-label results provides evidence that the observed classification performance was not attributable simply to chance associations.

The majority-class baseline provided a complementary assessment of the effect of class imbalance. Although this baseline achieved an overall accuracy of 0.2865 by assigning samples to the dominant interaction subtype, its balanced accuracy was only 0.0164, with a macro F1-score of 0.0073 and an MCC of 0.0000. The considerably stronger class-balanced performance of the machine-learning classifiers therefore indicates that their predictive behavior cannot be explained solely by exploitation of the dominant interaction class.

An important methodological consideration concerns the 423 canonical drug pairs associated with more than one documented interaction subtype. Detailed auditing showed that 363 of these pairs had multiple subtype annotations belonging to the same PK/PD branch, whereas 60 had annotations spanning both PK and PD branches. Together, these multi-annotated pairs represented only 0.1923% of unique Level 2 pairs. Because the present Level 2 task was formulated as single-label multiclass classification, individual pair–subtype associations were retained as separate labeled records, while canonical pair grouping ensured that all annotations associated with the same drug pair remained within the same data partition. The present formulation should therefore be interpreted as classification of documented pair–subtype associations rather than as a formal multilabel prediction framework. A dedicated multilabel formulation would provide a more natural representation of drug pairs associated with multiple simultaneous interaction mechanisms and represents a potential extension of the framework.

The adoption of canonical pair-disjoint partitioning eliminated direct overlap of canonical drug pairs between the training and test partitions and ensured that repeated, reversed, or multiply annotated instances of the same pair remained within a single partition. This provides a more stringent evaluation than a conventional record-level random split with respect to direct pair duplication. However, pair-disjoint partitioning does not prevent individual drug identities from occurring in both training and test pairs. Additional drug-identity-disjoint analyses therefore examined generalization under more stringent conditions. Predictive performance decreased when test pairs contained one drug unseen during training and decreased further when both drugs were absent from the training data. This behavior demonstrates the increasing difficulty of extrapolating from previously represented drug identities to entirely unseen chemical entities and emphasizes the distinction between pair-level generalization and drug-level generalization.

Evaluation of the complete hierarchical pipeline further demonstrated the effect of error propagation between Level 1 and Level 2. Across 43,975 evaluable drug pairs, corresponding to 99.75% coverage of the pair-disjoint test set, the end-to-end Top-1 subtype accuracy was 57.67%, while the end-to-end Top-3 accuracy reached 62.30%. Importantly, when Level 1 routing was correct, conditional Level 2 subtype accuracy increased to 92.08% for Top-1 and 99.46% for Top-3 predictions. Of the evaluated pairs, 16,431 errors originated at Level 1, whereas among correctly routed pairs, 2182 subsequently missed the correct Top-1 subtype and only 149 missed the correct subtype within the Top-3 predictions. These findings indicate that errors in the complete hierarchical pipeline are strongly influenced by PK/PD routing at Level 1 and identify improvement of the first classification stage as an important direction for future development.

Hierarchical consistency was also explicitly assessed because unconstrained subtype ranking can generate Level 2 outputs that conflict with the mechanistic branch predicted at Level 1. Across the complete test set of 44,086 drug pairs, raw unconstrained Level 2 outputs showed Top-1 branch consistency of 57.30%, while complete Top-3 branch consistency was only 7.13%. After application of Level 1-constrained subtype selection, branch consistency reached 100% for both Top-1 and Top-3 outputs. Thus, the revised hierarchical implementation prevents PK/PD-incompatible subtypes from being reported downstream of the Level 1 decision. This constraint improves the internal mechanistic coherence of the prediction workflow, although it does not correct an erroneous PK/PD decision made at Level 1.

Comparison of Level 1 predictions with available lookup-derived PK/PD annotations provided a separate assessment of agreement on documented interactions. Across 33,741 unique documented test pairs, 23,491 predictions agreed with the reference PK/PD annotation, corresponding to an overall agreement of 69.62%, whereas 10,250 pairs were discordantly classified. Consequently, documented interactions were not uniformly reproduced by the predictive model. This finding is important for interpretation of the web platform: reference lookup information and machine-learning predictions represent distinct outputs and should not be considered interchangeable. A documented interaction retrieved from the reference resource therefore remains visible independently of whether the model-generated PK/PD prediction agrees with its annotation.

Beyond model evaluation, DDI-HierPred was implemented as a web-based platform to facilitate practical exploration of the hierarchical workflow. The platform combines a documented-interaction lookup module with model-based prediction, enabling available reference information to be displayed separately from predictions generated by Level 1 and Level 2. It supports single-pair prediction, batch screening, confidence-score reporting, constrained Top-3 subtype ranking, and downloadable results. Importantly, the numerical values displayed by the platform are reported as model confidence scores rather than calibrated probabilities. For Level 1, the Linear SVM decision-function output is transformed for display without probability calibration, while Level 2 scores are retained on the original classifier output scale without renormalization after branch-incompatible subtypes are excluded. These scores should therefore be interpreted as indicators of model ranking or confidence rather than estimates of clinical probability.

The representative application to ten drug pairs illustrates the behavior of the revised platform following implementation of Level 1-constrained subtype selection. Reference lookup and model-generated prediction are presented independently, allowing documented interaction information to be inspected alongside, but not replaced by, the hierarchical prediction. For each evaluated pair, Level 2 Top-1 and Top-3 predictions were restricted to interaction subtypes belonging to the PK or PD branch selected by Level 1. These examples therefore illustrate the operational behavior of the deployed workflow rather than constituting an independent validation dataset. Similarly, predictions generated for pairs not retrieved from the lookup resource should be regarded as computational hypotheses for exploratory screening rather than evidence of previously undocumented clinical interactions.

Several limitations should therefore be considered when interpreting the present findings. First, although canonical pair-disjoint partitioning eliminates direct pair overlap, individual drug identities may still be represented across partitions, and the drug-disjoint analyses demonstrate a substantial reduction in performance under more stringent extrapolation conditions. Second, the hierarchical architecture propagates Level 1 errors to downstream subtype prediction, as demonstrated by the difference between conditional Level 2 performance and actual end-to-end performance. Third, the current Level 2 formulation is single-label and does not explicitly model simultaneous interaction subtypes for multi-annotated drug pairs. Fourth, the reported confidence scores are not probability-calibrated and should not be interpreted as quantitative estimates of clinical risk. Finally, additional validation using completely independent external DDI datasets would be required to establish generalizability beyond the data sources and drug space evaluated in the present study.

Overall, DDI-HierPred provides a computational framework for organizing DDI prediction into broad mechanistic classification followed by constrained interaction-subtype ranking. The present results support the feasibility of this hierarchical formulation while also demonstrating that performance depends strongly on drug familiarity and on correct Level 1 routing. Accordingly, the platform is intended as a research-oriented computational screening and hypothesis-generation tool rather than a substitute for curated interaction resources, experimental DDI assessment, clinical evidence, or professional decision-making.

In addition, the Level 2 algorithm comparison was performed using the established pair-disjoint test partition because an independent validation partition had not been retained during the original Level 2 model-development workflow. This may introduce model-selection bias, and the reported Level 2 performance should therefore be interpreted with this limitation in mind. Future studies should employ a predefined pair-disjoint train/validation/test design in which model selection is completed exclusively on the validation partition before final evaluation on an untouched test set.

## 5. Conclusions

In this study, DDI-HierPred was developed as a web-based hierarchical machine-learning framework for drug–drug interaction prediction using Morgan fingerprint-based drug-pair representations. The framework combines Level 1 pharmacokinetic/pharmacodynamic (PK/PD) classification using Linear SVM with Level 2 constrained interaction subtype prediction using an XGBoost multiclass classifier.

The Level 1 model achieved an accuracy of 0.8661 and ROC-AUC of 0.9380 on the primary test set, whereas the Level 2 model showed high balanced accuracy, macro F1-score, and Top-3 accuracy across 61 interaction subtype classes. However, when the complete hierarchical pipeline was evaluated using predicted Level 1 routing, performance decreased to an accuracy of 0.5767, balanced accuracy of 0.3573, and macro F1-score of 0.4379. Generalization also decreased under drug-identity-disjoint evaluation, with mean accuracies of 0.6578 ± 0.0196 for one-unseen-drug pairs and 0.4559 ± 0.0288 for fully drug-disjoint pairs. These findings demonstrate that the strong standalone and pair-disjoint results should not be interpreted as equivalent to performance on previously unseen drug identities and identify upstream routing and unseen-drug generalization as important limitations of the current framework. Y-randomization analyses further supported the robustness of both classification levels, indicating that the observed predictive performance was not attributable to chance label associations.

The implementation of DDI-HierPred as a publicly accessible web platform enables documented interaction lookup, single-pair prediction, batch screening, confidence score reporting, constrained subtype prediction, ranked Top-3 interaction subtype predictions, and downloadable results. Overall, DDI-HierPred provides a research-oriented computational platform for drug–drug interaction screening and hypothesis generation. Future work should focus on validation using independent external DDI datasets, improving Level 1 generalization and end-to-end hierarchical performance, expansion of underrepresented interaction subtype classes, and the incorporation of explainable artificial intelligence approaches to improve the interpretability of individual predictions.

## Figures and Tables

**Table 1:** Dataset provenance and record counts across the DDI-HierPred preprocessing workflow.

Processing Stage	Records/Pairs	Interpretation
Initial DDI collection	1,420,072	DDI records before structural-coverage filtering
PK/PD-annotated dataset	1,355,205	Records assigned to PK or PD for Level 1
Both-drug SMILES available	996,112	Interaction records with valid structural representation for both drugs
Final Level 2 canonical dataset	220,425	Unique canonical drug pairs retained for Level 2 after subtype preprocessing and filtering

**Table 2:** End-to-end comparison of flat Level 2 classification, oracle hierarchical routing, and predicted hierarchical routing on the common Level 2 evaluation subset (*n* = 43,975).

Model Configuration	Accuracy	Balanced Accuracy	Macro F1	MCC	Top-3 Accuracy
Flat 61-class model	0.8712	0.9086	0.9114	0.8507	0.9867
Oracle hierarchical routing	0.9243	0.9374	0.9378	0.9127	0.9935
Predicted hierarchical routing	0.5767	0.3573	0.4379	0.4968	0.6230

**Table 3:** Reduction in interaction subtype classes during Level 2 dataset preparation.

Dataset Refinement Step	Number of Interaction Subtypes
Original DeepDDI interaction subtypes	113
Retained after filtering	61
Removed due to insufficient representation	52

**Table 4:** Validation performance comparison of Level 1 PK/PD classification models.

Model	Accuracy	Balanced Accuracy	Macro F1	ROC-AUC	MCC
Linear SVM	0.8683	0.8674	0.8673	0.9418	0.7347
Logistic Regression	0.8632	0.8618	0.8621	0.9383	0.7241
Random Forest	0.7948	0.7840	0.7869	0.8904	0.5927
XGBoost	0.7963	0.7894	0.7917	0.8827	0.5897

**Table 5:** Confusion matrix of the final Linear SVM Level 1 model on the test set.

Actual/Predicted	Predicted PK	Predicted PD
Actual PK	3908	657
Actual PD	682	4753

**Table 6:** Y-randomization results for the Level 1 Linear SVM model.

Metric	Randomized Mean	Randomized SD
Accuracy	0.5139	0.0079
F1_PD	0.5977	0.0074
ROC-AUC	0.5014	0.0106
MCC	−0.0008	0.0165

**Table 7:** Pair-disjoint dataset audit.

Metric	Value
Unique drug pairs in training set	175,945
Unique drug pairs in test set	43,987
Shared canonical pairs	0
Pair leakage	0%
Unique drugs in training set	1828
Unique drugs in test set	1732
Shared drugs	1716
Test-set drug overlap	99.08%

**Table 8:** Level 2 predictive performance under progressively stringent generalization settings. Drug-identity-disjoint results are reported as mean ± standard deviation across five repeated splits.

Evaluation Setting	Accuracy	Balanced Accuracy	Macro F1	MCC	Top-3 Accuracy
Pair-disjoint	0.8712	0.9087	0.9115	0.8507	0.9868
One-unseen-drug	0.6578 ± 0.0196	0.6360 ± 0.0284	0.6825 ± 0.0269	0.5977 ± 0.0208	0.8722 ± 0.0079
Fully drug-disjoint	0.4559 ± 0.0288	0.3387 ± 0.0543	0.3717 ± 0.0655	0.3380 ± 0.0268	0.7349 ± 0.0135

Values for the one-unseen-drug and fully drug-disjoint settings represent mean ± SD across five repeated splits. Pair-disjoint values correspond to the primary fixed train/test partition used for model selection and subsequent analyses.

**Table 9:** Performance comparison of Level 2 classifiers and the majority-class baseline using the pair-disjoint test set.

Model	Accuracy	Balanced Accuracy	Macro F1	MCC	Top-3 Accuracy
XGBoost	0.8712	0.9087	0.9115	0.8507	0.9868
Random Forest	0.8823	0.8406	0.8740	0.8635	0.9900
SGD Logistic Regression	0.8667	0.7975	0.8301	0.8452	0.9838
Majority-class baseline	0.2865	0.0164	0.0073	0.0000	0.5771

**Table 10:** Y-randomization results for the Level 2 XGBoost model.

Metric	Original Model	Randomized Mean
Accuracy	0.8712	0.2864
Balanced Accuracy	0.9087	0.0164
Macro Precision	0.9181	0.0125
Macro Recall	0.9087	0.0164
Macro F1-score	0.9115	0.0074
Weighted F1-score	0.8700	0.1283
MCC	0.8507	0.0023
Top-3 Accuracy	0.9868	0.5765
Multiclass Log Loss	0.4868	2.7010

**Table 11:** Branch consistency of Level 2 predictions across the complete test set (*n* = 44,086).

Level 2 Prediction	Raw Unconstrained Output	Level 1-Constrained Output
Top-1 branch consistency	57.30%	100%
Top-3 branch consistency	7.13%	100%

**Table 12:** Agreement between Level 1 predictions and lookup-derived PK/PD annotations for unique documented test pairs (*n* = 33,741).

Lookup PK/PD Class	Predicted PD	Predicted PK	Total
PD	14,447	5607	20,054
PK	4643	9044	13,687
Total	19,090	14,651	33,741

**Table 13:** End-to-end performance of the hierarchical DDI-HierPred pipeline.

Metric	Result
Evaluated drug pairs	43,975
Coverage of pair-disjoint test set	99.75%
Level 1 accuracy	62.64%
End-to-end Top-1 subtype accuracy	57.67%
End-to-end Top-3 subtype accuracy	62.30%
Top-1 subtype accuracy given correct Level 1	92.08%
Top-3 subtype accuracy given correct Level 1	99.46%

**Table 14:** Representative outputs generated by the updated DDI-HierPred web platform.

Drug Pair	Lookup Status	Level 1	Level 1 Score	Level 2 Top-1 Subtype	Level 2 Score
Ethotoin—DB00755	Not found	PK	0.6454	DB00755 may increase the thrombogenic activities of Ethotoin.	0.1694
Ethotoin—Dolasetron	Documented	PD	0.7130	Increased adverse effects	0.0056
DB00755—Tetracycline	Documented	PK	0.5533	Decreased therapeutic efficacy	0.1942
Dolasetron—Tetracycline	Not found	PD	0.8336	Increased adverse effects	0.0360
Meropenem—Irinotecan	Not found	PK	0.7995	Increased serum concentration	0.2524
Irinotecan—Mometasone	Documented	PD	0.9912	Increased adverse effects	0.2595
Mometasone—Olopatadine	Not found	PD	0.9205	Increased adverse effects	0.3290
Olopatadine—Alprostadil	Not found	PK	0.9965	Decreased therapeutic efficacy	0.8431
Alprostadil—DB00772	Not found	PK	0.9455	DB00772 may increase the antiplatelet activities of Alprostadil.	0.2897
DB00772—Oxcarbazepine	Not found	PD	0.6948	Increased adverse effects	0.0185

For brevity, only the highest-ranked Level 2 subtype is reported in the table. The complete Top-3 predictions and their corresponding confidence scores are displayed in the DDI-HierPred web interface.

## Data Availability

The web application is available at: https://huggingface.co/spaces/Prof-ouassaf/DDI-Hierarchical-Predictor, accessed on 16 June 2026. The datasets, trained models, and curated lookup resources generated during this study are publicly available at Zenodo: https://doi.org/10.5281/zenodo.21788445.

## Supplementary Materials

The following supporting information can be downloaded at: https://www.mdpi.com/article/10.3390/pharmaceutics18081042/s1.
