# Supplementary material for: DDI-HierPred: An Artificial Intelligence-Based Hierarchical PK/PD Platform for Drug–Drug Interaction Prediction
Source: Pharmaceutics. 2026 Aug 21;18(8):1042. doi: 10.3390/pharmaceutics18081042 (PMC13516539; doi:10.3390/pharmaceutics18081042)
Supplement: Supplementary file 1 [file pharmaceutics-18-01042-s001.zip › pharmaceutics-4450264-supplementary.pdf]

Supplementary Material

DDI-HierPred: An Artificial Intelligence-Based Hierarchical PK/PD Platform for Drug–Drug Interaction Prediction

Mebarka OUASSAF[1]*, and Bader Y. ALHATLANI[2]*

[1]Group of Computational and Medicinal Chemistry, LMCE Laboratory, University of Biskra, Biskra (07000), Algeria; nouassaf@univ-biskra.dz

[2]Unit of Scientific Research, Applied College, Qassim University, Buraydah 52571, Saudi Arabia; balhatlani@qu.edu.sa

* Correspondence: balhatlani@qu.edu.sa (B.Y.A.); nouassaf@univ-biskra.dz (M.O.)

Table S1. Composition of the five repeated drug-identity-disjoint evaluation splits.

**Seed**	**Training pairs**	**Unique drugs**	**One-unseen pairs**	**Unique drugs**	**Fully disjoint pairs**	**Unique drugs**	**Subtypes (Train/One/Fully)**
10	140,903	1,474	70,038	1,754	8,577	354	61/61/54
11	141,507	1,468	69,559	1,715	8,452	342	61/61/52
12	141,113	1,450	69,817	1,770	8,588	343	61/61/49
14	141,855	1,465	69,345	1,720	8,318	335	61/61/53
17	138,363	1,466	71,844	1,746	9,311	342	61/61/52
